# Delivery mode and subsequent birth rate: A nationwide register‐based analysis in Finland

**DOI:** 10.1002/ijgo.15982

**Published:** 2024-10-27

**Authors:** Matias Vaajala, Rasmus Liukkonen, Ville M. Mattila, Maiju Kekki, Ilari Kuitunen

**Affiliations:** ^1^ Faculty of Medicine and Life Sciences University of Tampere Tampere Finland; ^2^ Department of Orthopaedics and Traumatology Tampere University Hospital Tampere Tampere Finland; ^3^ Department of Obstetrics and Gynecology Tampere University Hospital Tampere Finland; ^4^ Center for Child, Adolescent and Maternal Health Research, Faculty of Medicine and Health Technology Tampere University Tampere Finland; ^5^ Department of Pediatrics Mikkeli Central Hospital Mikkeli Finland; ^6^ Institute of Clinical Medicine and Department of Pediatrics University of Eastern Finland Kuopio Finland

**Keywords:** birth rate, delivery mode, epidemiology, obstetrics

## Abstract

**Objective:**

The study aimed to calculate the subsequent birth rate for different delivery modes, comparing them with spontaneous vaginal deliveries, using a comprehensive nationwide high‐quality registry.

**Methods:**

Data from the National Medical Birth Register (MBR) were used to evaluate the birth rate after different delivery modes. All first deliveries for a mother during the years 2004 to 2016 were included. For these women, all second pregnancies from the MBR during the years 2004 to 2018 were retrieved and combined with the data of the first deliveries. A Cox regression model was used to evaluate the risk for the second pregnancy after giving birth the first time. The results were interpreted with adjusted hazard ratios (aHRs) and 95% confidence intervals (CIs).

**Results:**

A total of 375 619 women with a first and second pregnancy leading to birth were included in this study. Of these, a total of 50 579 women underwent assisted vaginal delivery, 50 429 had an unplanned cesarean section (CS), 22 021 had elective CS, and 252 593 had spontaneous vaginal delivery as a mode of delivery in their first pregnancy. Women with assisted vaginal delivery (aHR, 1.23 [CI, 1.21–1.24]) and unplanned CS (aHR, 1.03 [CI, 1.02–1.05]) had higher birth rates after the first birth, and women with elective CS as a mode of delivery had lower birth rates (aHR, 0.86 [CI, 0.84–0.88]) when compared with women who had spontaneous vaginal delivery.

**Conclusion:**

The findings of this study indicate that the CS operation itself is not the only cause of the observed lower birth rate; rather, there are underlying factors that have a greater impact on birth rates.

## Synopsis

Cesarean section itself is not the only cause of the observed lower birth rate; there are underlying factors that have a greater affect on birth rates.

## 1 INTRODUCTION

Research on the relationship between mode of delivery, especially cesarean section (CS), and subsequent fertility is important, as the rates of CS have increased rapidly during the past decades.^1^, ^2^ Indeed, the global average annual CS rate has more than tripled over the past 25 years, increasing from 6.7% in 1990 to 21.1% in 2015.^1^, ^2^

Several studies have explored the association between the mode of delivery and subsequent birth rate. The latest investigation examining this association was a prospective study using a sample of 3006 women.^3^ In that study, women who underwent CS as a mode of delivery were found to have a lower birth rate when compared with women who had vaginal deliveries during a 3‐year follow‐up.^3^ Furthermore, many previous studies have also reported that women who undergo CS as a mode of delivery have lower subsequent fertility.^4^, ^5^, ^6^, ^7^, ^8^, ^9^ The latest systematic review in 2020 reported a 13% lower probability of subsequent fertility in women who delivered by emergency cesarean birth, a 14% lower probability by elective cesarean birth, a 39% by maternal‐requested cesarean birth, and a 2% instrumental vaginal delivery when compared with women with spontaneous vaginal deliveries.^10^

In addition, findings on whether CS actually decreases the subsequent birth rate have been contradictory. Various explanations have been proposed, ranging from placental bed disruption or pelvic adhesions to women's reproductive choices.^11^, ^12^, ^13^ However, the latest study found that the lower rate of subsequent childbearing after cesarean delivery was primarily associated with failure to conceive and was not voluntary.^3^ In addition, the women whose first birth was by CS did not differ from those who delivered vaginally on any psychosocial measures, including fear of childbirth, depression, social support, or plans to have a second child.^3^

As the association between different modes of delivery and subsequent birth rate has only been moderately studied and a history of methodological deficits and previous contradictory results exist, this study aimed to calculate the birth rate among different modes of delivery compared with spontaneous vaginal deliveries using high‐quality nationwide registers in Finland.

## 2 MATERIALS AND METHODS

This nationwide retrospective cohort study used data from the National Medical Birth Register (MBR) to assess birth rates among women who experienced various modes of delivery, compared with those who had a spontaneous vaginal delivery. The MBR is overseen by the Finnish Institute for Health and Welfare, and the study period spanned from January 1, 2004, to December 31, 2018.

The MBR contains data on pregnancies, delivery statistics, and the perinatal outcomes of all births with a birth weight of ≥500 g or a gestational age of ≥22^+0^ weeks. The MBR has high coverage (nearly 100%) and quality.^14^, ^15^ All women who had their first delivery during the years 2004–2016 were included. For these women, all data on second pregnancies leading to births during the years 2004 to 2018 were retrieved from the MBR and combined with the data of the first deliveries. These cohorts were then used to evaluate the association between delivery mode in the first pregnancy and the subsequent birth rate after the first birth.

**FIGURE 1:**
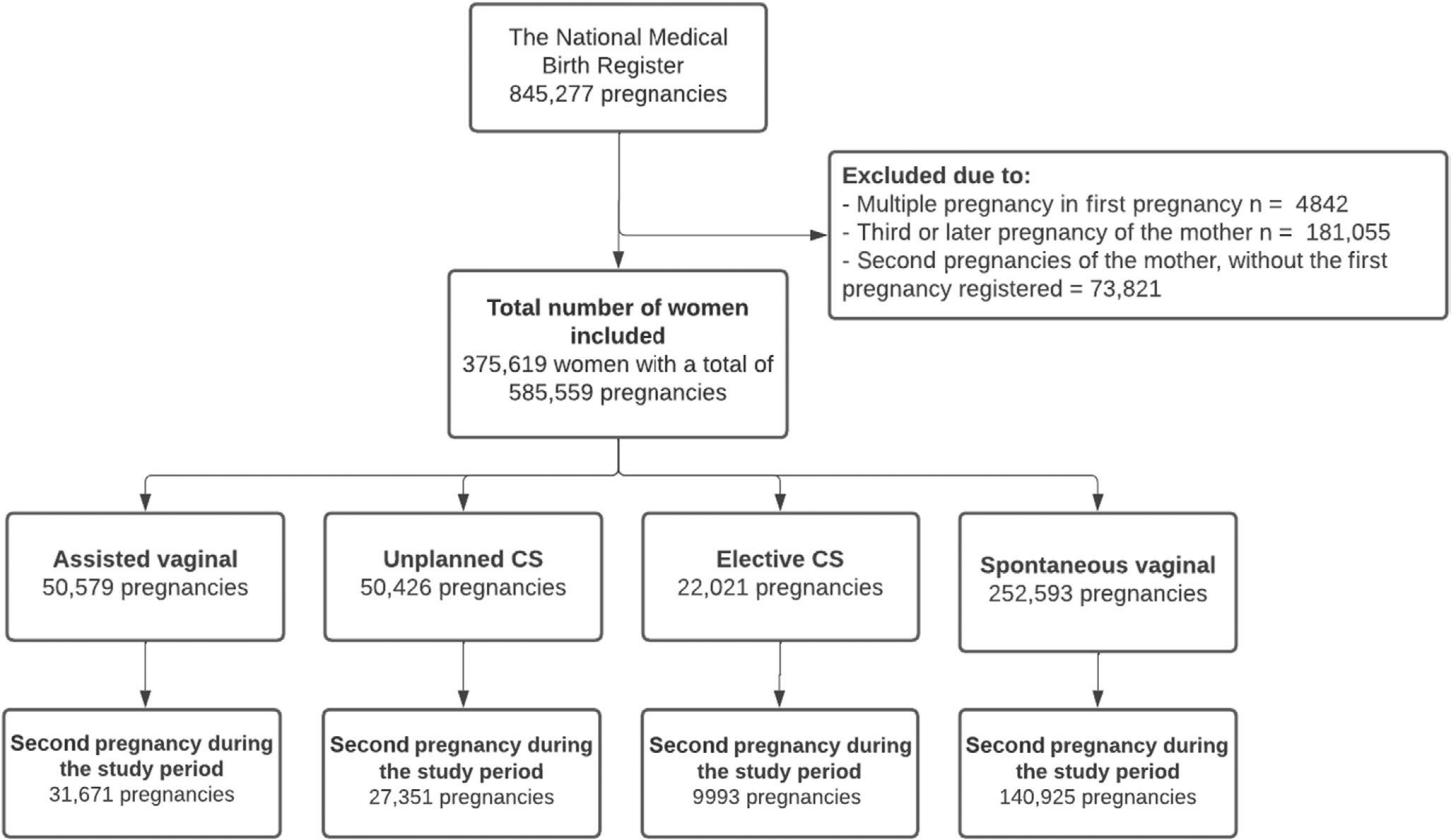
Flowchart depicting the process used to divide the study population into groups. CS, cesarean section.

The birth rate after the first pregnancy was compared between different modes of delivery. The mode of delivery was divided into four groups: assisted vaginal delivery (including vacuum and forceps), unplanned CS (including urgent and emergency CS), elective CS, and spontaneous vaginal delivery, which was used as a reference group. Spontaneous vaginal breech deliveries were also placed in the spontaneous vaginal delivery group. The first pregnancy had to be a singleton pregnancy, as multiple pregnancies may influence whether the woman gets pregnant again. Only pregnancies ending in delivery after gestational week 21 + 6 were included, as data on pregnancies ending in miscarriage or induced abortions were not available in our data. Stillbirths were, however, also included. The process used to form the study groups and the compared groups is shown as a flowchart in Figure 1.

To address residual confounding, we gathered information on the background of the women at the time of the first pregnancy using the variables found in the MBR. The age of the mother, year of the pregnancy, marital and socioeconomic status, smoking status, maternal gestational diabetes, maternal body mass index (BMI) as a categorized variable, prior miscarriages and abortions, neonatal sex, preterm deliveries (<37 + 0 weeks), low birth weight (<2500 g), and need for neonatal intensive care are the variables routinely registered in the MBR. Socioeconomic status was categorized into five classes (high, mid, low, miscellaneous, and missing) using the classification shown in Table S1. Maternal BMI was categorized into underweight (BMI <18.5 kg/m^2^), normal weight (18.5 kg/m^2^ ≤BMI <25.0 kg/m^2^), overweight (25.0 kg/m^2^ ≤BMI < 30.0 kg/m^2^), and obese (BMI ≥30.0 kg/m^2^) using the World Health Organization's classification.^16^ Gestational diabetes was diagnosed using the 75 g 2‐h oral glucose tolerance test.

### 2.1 Statistical analysis

Continuous variables are presented as means with standard deviations (SDs). The categorical variables are presented as absolute numbers and percentages with *p* values. The *p* values for categorical variables were calculated using χ^2^ tests, and for continuous variables using Student *t*‐tests. The Cox regression model was used to evaluate the risk for the second pregnancies leading to birth after the first birth in women who underwent different modes of delivery in relation to the reference women who had a spontaneous vaginal delivery in their first pregnancy. The results are presented as adjusted hazard ratios (aHRs) and 95% confidence intervals (CIs). The proportional hazards assumption was tested using Schoenfeld residuals and the assumption was unviolated. The start of the follow‐up was the date of giving birth in the first pregnancy. The first birth had to have occurred during the years 2004 to 2016, as women who gave birth later had no time to become pregnant again in our data. The end point of the follow‐up was the start of the second pregnancy, the end date of our study period (December 31, 2018), or the date when the mother turned 50 years old, as our data include only women aged 15–49 years. The model was adjusted by categorized maternal age, as age influences the decision and physical ability to conceive again, categorized maternal BMI,^17^ and the year of the pregnancy, given that the birth rates have shown a decreasing trend during the past decades in Finland,^18^ which affects the available follow‐up time. In addition, based on our results (the proportional differences between different delivery modes, and between the patients who had their second birth and those who did not), the model was adjusted by gestational diabetes, socioeconomic status, smoking status, and prior miscarriages and induced abortions, as these variables differed markedly between those who had their second pregnancy and those who did not. In addition, Kaplan–Meier survival curves between the groups were generated to visualize the birth rates. The cut point for statistical significance was 0.05. The results of this study are reported according to STROBE (Strengthening the Reporting of Observational Studies in Epidemiology) guidelines.^19^ Statistical analyses were performed using R version 4.0.3 for Windows (R Foundation for Statistical Computing).

### 2.2 Ethics

All procedures were conducted in accordance with Finnish regulations. The ethical committee of Tampere University Hospital waived the need for ethical review for all retrospective studies using routinely collected healthcare data, in compliance with the Medical Research Act (488/1999) and the Patient Rights Act (785/1992). As per Finnish regulations (Act on the Secondary Use of Health and Social Data, 552/2019), informed written consent was not required becaause of the retrospective, register‐based nature of the study, and no patients were contacted. Permission to use the data was granted by Findata following an evaluation of the study protocol (permission number: THL/1756/14.02.00/2020).

## 3 RESULTS

A total of 375 619 women were included in this study. Of these, 50 579 underwent assisted vaginal delivery (vacuum, *n* = 50 353; forceps, *n* = 226), 50 429 underwent unplanned CS, 22021 underwent elective CS, and 252 593 had a spontaneous vaginal delivery (breech, *n* = 2667) as a mode of delivery in their first pregnancy. Subsequently, 31 671 (62.6%) women who underwent assisted vaginal delivery, 27 351 (54.2%) who underwent unplanned CS, 9993 (45.4%) who underwent elective CS, and 140 925 (55.8%) who had a spontaneous vaginal delivery became pregnant again and delivered during the study period (*p* < 0.001) (Table 1).

**TABLE 1:** Background information and factors associated with mode of delivery.

Total number of women	Assisted vaginal delivery	%	Unplanned CS 50 429		Elective CS 22 021 *n*	%	Spontaneous vaginal (control group) 252 593 *n*	%	*p*‐value
	50 579		*n*	%					
	Number								
Age at first birth, mean (SD), y	28.9 (5.2)		29.6 (5.5)		30.4 (5.6)		27.8 (5.2)		<0.001
< 20	1517	3.0	1264	2.5	508	2.3	12 207	4.8	<0.001
20–24	8944	17.7	8134	16.1	2888	13.1	58 166	23.0	
25–29	17 643	34.9	15 865	31.5	6401	29.1	90 420	35.8	
30–34	15 132	29.9	15 622	31.0	6997	31.8	64 914	26.1	
35–39	5993	11.9	7541	15.0	4029	18.3	22 744	9.0	
≥ 40	1350	2.7	2003	4.0	1198	5.4	4127	1.6	
Year of pregnancy									<0.001
Between 2004 and 2007	11 603	22.9	12 659	25.1	7809	35.5	83 743	33.2	
Between 2007 and 2010	12 029	23.8	12 128	24.1	4984	22.6	57 079	22.6	
Between 2010 and 2013	11 909	23.6	11 619	23.0	4246	19.3	51 135	20.3	
Between 2013 and 2016	15 038	29.7	14 023	27.8	4982	22.6	60 621	24.0	
Marital status									<0.001
Never married	25 657	50.7	25 751	51.1	10 420	47.3	129 191	51.2	
Unknown	71	0.1	80	0.2	16	<0.1	556	<0.1	
Socioeconomic status									<0.001
High	8693	17.2	8336	16.5	4057	18.4	39 313	15.6	
Mid	14 895	29.5	15 501	30.7	7112	32.3	73 505	29.1	
Low	6575	13.0	7095	14.1	2995	13.6	35 999	14.3	
Miscellaneous	6632	13.1	6186	12.3	2592	11.8	39 677	15.7	
Unknown	13 784	27.3	13 311	26.4	5265	23.9	64 084	25.4	
Smoking status smoker	7988	15.8	8691	17.2	3509	15.9	45 618	18.1	<0.001
Maternal gestational diabetes	5677	11.2	7644	15.2	3025	13.7	24 539	9.7	<0.001
Maternal BMI, mean (SD), kg/ m^2^	23.8 (4.5)	17.1	25.3 (5.3)		24.7 (5.1)		23.7 (4.5)		<0.001
Underweight (BMI <18.5)	2057	4.1	1351	2.7	766	3.5	10 842	4.3	
Normal weight (18.5 ≤ BMI < 25.0)	32 480	64.2	26 787	53.1	12 281	55.8	158 808	62.9	
Overweight (25.0 ≤ BMI < 30.0)	4600	9.1	11 482	22.8	4600	20.9	45 501	18.0	
Obesity class I (30.0 ≤ BMI < 35.0)	1811	3.6	5087	10.1	1811	8.2	15 286	6.1	
Obesity class II (35.0 ≤ BMI < 40.0)	683	1.4	1924	3.8	687	3.1	4763	1.9	
Obesity class III (BMI ≤40.0)	303	0.6	976	1.9	303	1.4	1864	0.7	
BMI unknown	8645		2822	5.6	1573	7.1	18 293	7.2	
Prior miscarriages	7139	14.1	8522	16.9	3950	17.9	36 947	14.6	<0.001
Prior induced abortions	5484	10.8	6274	12.4	2848	12.9	30 563	12.1	<0.001
Neonatal sex boy	28 460	56.3	27 930	55.4	10 749	48.8	124 990	49.5	<0.001
Preterm delivery (<37 + 0 weeks)	1267	2.5	5591	11.1	1321	6.0	11 008	4.4	<0.001
Low birth weight (<2500 g)	877	1.7	4977	9.9	1163	5.3	7691	3.0	<0.001
Neonatal intensive care unit	7062	14.0	12 855	25.5	3206	14.6	23 086	9.1	<0.001
Time difference between pregnancies, mean (SD), y	2.1 (1.5)		2.2 (1.6)		2.3 (1.6)		2.1 (1.6)		<0.001
Follow‐up time available, mean (SD)	8.5 (3.7)		8.8 (3.7)		9.6 (3.8)		9.4 (3.8)		<0.001
Became pregnant again									<0.001
Total	31 671	62.6	27 351	54.2	9993	45.4	140 925	55.8	<0.001
During 1‐year follow‐up	6624	13.1	5151	10.2	1666	7.6	31 608	12.5	<0.001
During 3‐year follow‐up	25 676	50.8	21 426	42.5	7780	35.3	113 924	45.1	<0.001
During 5‐year follow‐up	29 921	59.2	25 486	50.5	9278	42.1	131 951	52.2	<0.001
During 7‐year follow‐up	31 104	61.5	26 750	53.0	9751	44.3	137 876	54.6	<0.001
During 9‐year follow‐up	31 270	61.8	27 155	53.9	9925	45.1	138 831	55.0	<0.001

*Note*: Patients with assisted vaginal delivery, unplanned cesarean section (CS), and elective CS were compared with a control group consisting of spontaneous vaginal deliveries. The rates are presented with *p* values. BMI, body mass index; SD, standard deviation.

The mean age for women in the spontaneous vaginal delivery group was younger (mean, 27.8 years; SD, 5.2) than women who underwent assisted vaginal delivery (mean, 28.9 years; SD, 5.2), unplanned CS (mean, 29.6 years; SD, 5.5), and elective CS (mean, 30.4 years; SD, 5.6) (*p* < 0.001). The highest rate of smoking was found in the spontaneous vaginal delivery group (18.1%) (*p* < 0.001). The highest rate of diagnosed gestational diabetes was found among women who underwent elective CS as a mode of delivery (13.7%) (*p* < 0.001). Women with elective CS had the highest rate of prior miscarriages (17.9%) (*p* < 0.001). Women with elective CS as the mode of delivery had the longest time difference between pregnancies (mean, 2.3 years; SD, 1.6) (*p* < 0.001). Women who underwent elective CS also had a notably lower rate of second pregnancies leading to births after 1‐year follow‐up (7.6%) than women in the other study groups. Moreover, the rate remained the lowest throughout the whole study period (*p* < 0.001) (Table 1).

When analyzing the factors associated with the outcome of our study (the second birth), women who had their second birth during our study period were older than women who did not give birth again (mean age, 27.0 vs. 30.0 years). In addition, a higher rate of smokers was observed among those who did not have their second pregnancy during our study period (19.3% vs. 16.1%, *p* < 0.001). A lower rate of women who had their second birth had gestational diabetes in the first pregnancy than those who did not have their second birth (9.3% vs. 12.9%, *p* < 0.001). A higher rate of women who had their second pregnancies leading to birth had normal‐weight BMI than women who did not give birth (64.1% vs. 57.8%, *p* < 0.001). Also, a history of induced abortions and miscarriages was more common among women who did not have a second pregnancy (*p* < 0.001) (Table 2).

**TABLE 2:** Factors associated with second delivery during the study period.

Outcome in this study	No of second birth 165 672 *n*	Percentage	Second birth 209 947 *n*	Percentage	*p* value
Total number of women					
Age at first birth, mean (SD), y	30.0 (5.7)		27.0 (5.7)		<0.001
< 20	4679	2.8	10 817	5.2	<0.001
20–24	25 215	15.2	52 920	25.2	
25–29	46 937	28.3	83 398	39.7	
30–34	51 739	31.2	50 927	24.3	
35–39	29 155	17.6	11 154	53.1	
≥ 40	7947	4.8	731	0.3	
Year of pregnancy					<0.001
Between 2004 and 2007	60 125	36.3	55 700	26.5	
Between 2007 and 2010	30 363	18.3	55 858	26.6	
Between 2010 and 2013	26 293	15.9	52 616	25.1	
Between 2013 and 2016	48 891	29.5	45 773	21.8	
Marital status					<0.001
Never married	82 145	49.6	108 876	51.9	
Unknown	351	0.2	372	0.2	
Socioeconomic status					<0.001
High	26 554	16.0	33 846	16.1	
Mid	49 582	29.9	61 434	29.3	
Low	25 395	15.3	27 270	13.0	
Miscellaneous	21 479	13.0	33 612	16.0	
Unknown	42 662	25.8	53 785	25.6	
Smoking status smoker	31 943	19.3	33 863	16.1	<0.001
Maternal gestational diabetes	21 347	12.9	19 539	9.3	<0.001
Maternal BMI, mean (SD), kg/m^2^	24.3 (4.9)		23.7 (4.4)		<0.001
Underweight (BMI <18.5)	6458	3.9	8558	4.1	<0.001
Normal weight (18.5 ≤ BMI <25.0)	95 747	57.8	134 617	64.1	
Overweight (25.0 ≤ BMI <30.0)	32 888	19.9	38 088	18.1	
Obesity class I (30.0 ≤ BMI <35.0)	12 571	7.6	12 744	6.1	
Obesity class II (35.0 ≤ BMI <40.0)	4373	2.6	4018	1.9	
Obesity class III (BMI ≤40.0)	1949	1.2	1587	0.8	
BMI unknown	11 686	7.1	10 335	4.9	
Prior miscarriages	29 400	17.8	27 163	12.9	<0.001
Prior induced abortions	24 226	14.6	20 942	10.0	<0.001
Neonatal sex, male	84 481	51.0	107 657	51.3	0.082
Preterm delivery (<37 + 0 weeks)	8858	5.3	10 330	4.9	<0.001
Low birth weight (<2500 g)	6918	4.2	7791	3.7	<0.001
Neonatal intensive care unit	21 116	12.8	25 094	12.0	<0.001

*Note*: Patients with assisted vaginal delivery, unplanned cesarean section (CS), and elective CS were compared with a control group consisting of patients with spontaneous vaginal deliveries. The rates are presented with 95% confidence intervals (CIs).

Abbreviations: BMI, body mass index; SD, standard deviation.

**FIGURE 2:**
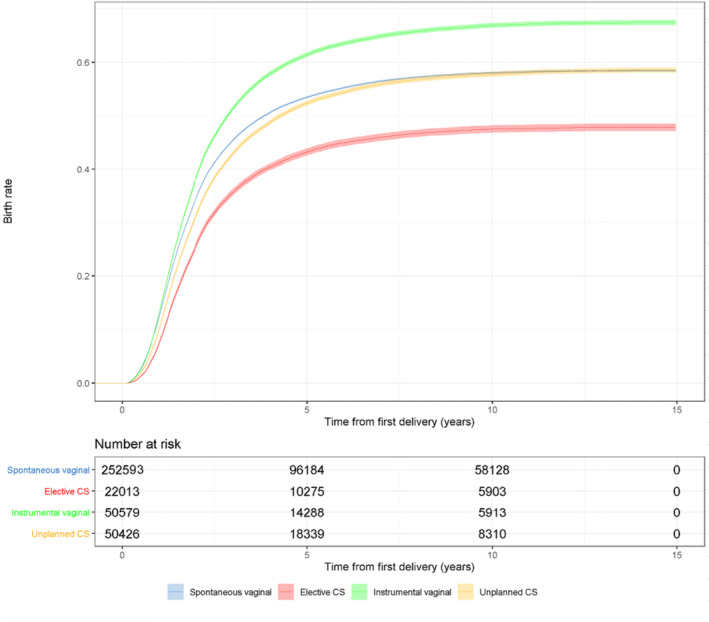
Kaplan–Meier survival curve with 95% confidence intervals for the event of women becoming pregnant again after their first delivery. Women with assisted vaginal delivery, unplanned cesarean section (CS), and elective CS were compared with women who had spontaneous vaginal deliveries (including breech).

Women who underwent elective CS as a mode of delivery had a notably lower proportion of second pregnancies leading to birth than women in the spontaneous vaginal delivery group (*p* < 0.001). The proportion of second pregnancies leading to birth in the elective CS group was 45.4% and 55.7% in the spontaneous vaginal delivery group (*p* < 0.001). Women who had assisted vaginal delivery or unplanned CS were more likely to become pregnant again after their first delivery (*p* < 0.001). Women who had elective CS were less likely to have a second pregnancy during the follow‐up (*p* < 0.001) (Figure 2).

Women who underwent assisted vaginal delivery (aHR, 1.23 [CI, 1.21–1.24]) and unplanned CS (aHR, 1.03 [95% CI, 1.02–1.05]) had a higher birth rate after their first delivery compared with the spontaneous vaginal delivery group. Women who had elective CS as a mode of delivery had a notably lower hazard for the second pregnancies leading to birth after the first delivery than those women in the spontaneous vaginal delivery group (aHR, 0.86 [95% CI, 0.84–0.88]) (Table 3). When all CS deliveries (unplanned and elective) were compared with spontaneous vaginal deliveries, the hazard for the second pregnancies leading to birth was similar to that with the elective CS (aHR, 0.86 [CI, 0.84–0.88]).

**TABLE 3:** AHRs with 95% CIs for the event of women becoming pregnant again after their first delivery.

Delivery mode	aHR* (95% CI)
Assisted vaginal delivery	1.23 (1.21–1.24)
Unplanned CS	1.03 (1.02–1.05)
Elective CS	0.86 (0.84–0.88)

*Note*: Women with assisted vaginal delivery, unplanned cesarean section (CS), and elective CS were compared with women who had spontaneous vaginal deliveries (including breech).

Abbreviations: aHR, adjusted hazard ratio; CI, confidence interval.

* Adjusted by categorized maternal age and body mass index, year of pregnancy, gestational diabetes, socioeconomic status, smoking status, prior miscarriages, and prior induced abortions.

## 4 DISCUSSION

The main finding of the present study is that women who underwent elective CS as a mode of delivery had a notably lower subsequent birth rate. However, the birth rate in women who underwent unplanned CS was not lower than the birth rate in women who had spontaneous vaginal deliveries. This indicates that the CS operation itself is not the only cause of the lower birth rate observed and that there are underlying factors that affect the birth rate. Furthermore, the birth rate was also not lower among women who underwent assisted vaginal delivery when compared with women who had spontaneous vaginal deliveries.

Several studies have examined the relationship between the mode of delivery and future birth rates. The most recent research on this topic was a prospective study involving a sample of 3006 women.^3^ This study found that women who opted for CS had a lower birth rate compared with those who had vaginal deliveries during a 3‐year follow‐up period.^3^ Furthermore, the majority of prior studies have indicated that women who undergo CS as their delivery method tend to exhibit lower subsequent fertility.^4^, ^5^, ^6^, ^7^, ^8^, ^9^, ^20^ However, when compared with our study, there were some limitations in the study designs of these previous studies, e.g. some of the studies compared only CS generally and vaginal deliveries, and the different types of CS were not analyzed separately. However, the latest study by Kjerulff et al. compared elective and unplanned CS separately with spontaneous vaginal deliveries, but, as there were no significant differences, the two cesarean delivery modes were combined.^3^ Also, a recent systematic review in 2020, which reported that in seven studies, emergency cesarean birth and elective cesarean birth were distinguished and compared with spontaneous vaginal deliveries, concluded that nonspontaneous vaginal delivery may be associated with a lower probability of subsequent fertility.^10^

According to previous systematic reviews and meta‐analyses, the methods and reporting of results in studies on this topic have been inconsistent, associated with significant weaknesses in study designs and analytical methods, and missing confounding factors such as maternal BMI.^7^ In our study, which included a large nationwide study sample of more than 70 000 cases of CS, the birth rate after unplanned CS was higher than the birth rate in the spontaneous vaginal delivery group. However, the birth rate was markedly lower among women who underwent elective CS. Interestingly, when using the same methods as those used in some of the previous studies (comparing all CS with vaginal deliveries), our data reveal a lower birth rate for women who underwent CS (Figure S1). These results should, therefore, raise doubts about the consistency of the methodology and the findings, as to whether the lower birth rate after CS is actually caused by the mode of delivery. Based on the finding that the subsequent birth rate was higher in a cohort of more than 50 000 women who underwent unplanned CS, it appears that the association between CS itself and lower birth rate might be caused even more by the underlying factors behind the CS. Because of the crude nature of our data, however, the reasons behind the lower birth rate, especially among women who underwent elective CS, remain unknown and should, therefore, be studied further using more precise data that include information on the reasons behind the lower birth rate.

A retrospective register‐based study in 2014 separately investigated the association between different types of CS and found that, especially after elective CS, the subsequent birth rate was lower when compared with vaginal deliveries.^7^ Another study that also analyzed the different types of CS found a slightly higher relative risk ratio for another pregnancy for all types of CS, with the risk ratio being lowest for elective CS.^21^ However, the number of studies that assess different types of CS is small, and the topic warrants further research. This is an important topic, as those factors that lead to elective CS can also serve to explain the lower subsequent birth rate. For example, even though psychological factors, such as fear of childbirth, are known to be strong indications for elective CS,^22^ they are, at the same time, possible indications for the decision not to get pregnant. According to a systematic review, the reasons for a maternal request for elective CS may vary, as fear of childbirth and emotional aspects may also influence the decision to request elective CS.^23^ In addition to these factors being indications for elective CS, they might also increase the threshold for conceiving again. A previous study conjectured that the lower rate of subsequent childbearing after cesarean delivery could be voluntary.^12^ Moreover, the results of the study indicate that the decreased birth rate observed after elective CS might be better explained by these social/psychological factors. It should be noted, however, that women in Finland who undergo delivery by CS are recommended to take 6–12 months to recovery between the CS procedure and the subsequent pregnancy, as the uterus requires time to heal from the surgical procedure. According to previous literature, the median interval between the birth of the first child and the beginning of the next pregnancy was 20 months for the CS group and 18 months for the reference group, comprising vaginal deliveries.^24^ No evidence of differences in interpregnancy intervals was found in that study.^24^ However, as the birth rate after unplanned CS is similar to that after spontaneous vaginal delivery, these findings could affect the results of the present study.

It should be noted, however, that elective and unplanned CS are usually not totally similar procedures. The elective nature of CS implies a deliberate decision‐making process, suggesting that women opting for this mode of delivery might have distinct characteristics or underlying health considerations compared with those with unplanned CS or the control group. This divergence could be influenced by various factors such as preexisting medical conditions, maternal age, or even socioeconomic factors. In addition, the elective nature of CS may involve specific medical interventions or conditions that impact subsequent fertility differently than unplanned CS.

The strengths of our study are the large nationwide register data used and the long study period, which allowed us to investigate the association between modes of delivery and subsequent birth rate. The register data used in our study are routinely collected in structured forms using national instructions, which ensures good coverage (over 99%) and reduces possible reporting and selection biases. However, the results of this study might differ from the results from other countries, as the CS rate in Finland is one of the lowest in the world and, therefore, the indications for CS might vary between countries.^25^ In addition, cultural differences in maternity care might lead to different kinds of results on this topic.

The main limitation of this study is the retrospective study design, which is why more precise information on the cause of the lower birth rate is missing. Furthermore, the possible date of death and migration of the women is not available in our data, making it impossible to identify women who were lost to follow‐up. In addition, only pregnancies leading to birth were included, as we had no information on miscarriages and induced abortions. Therefore, we do not information on the rate of women who tried to conceive a second child but were not able to give birth.

## 5 CONCLUSIONS

Women who undergo elective CS as a mode of delivery had a notably lower subsequent birth rate. However, when women who underwent unplanned CS were compared with women who had spontaneous vaginal deliveries, no lower birth rate was observed. This indicates that the CS operation itself is not the only cause of the lower birth rate observed and that there are underlying factors that affect the birth rate more. Therefore, further research is warranted.

## AUTHOR CONTRIBUTIONS

MV and RL wrote the initial manuscript. IK, VM, and MV undertook the study design. MK provided clinical expertise. Each author commented on the manuscript during the process and confirmed the final version to be submitted.

## FUNDING INFORMATION

This study has not received funding.

## CONFLICT OF INTEREST STATEMENT

The authors declare no conflict of interest.

## Supporting information


Figure S1.

Table S1.

## Data Availability

Research data are not shared.
