# Supplementary material for: Delivery mode and subsequent birth rate: A nationwide register‐based analysis in Finland
Source: Int J Gynaecol Obstet. 2024 Oct 27;168(3):1161–70. doi: 10.1002/ijgo.15982 (PMC11823355; doi:10.1002/ijgo.15982)
Supplement: Supplementary file 1 — Figure S1. [file IJGO-168-1161-s002.pdf]

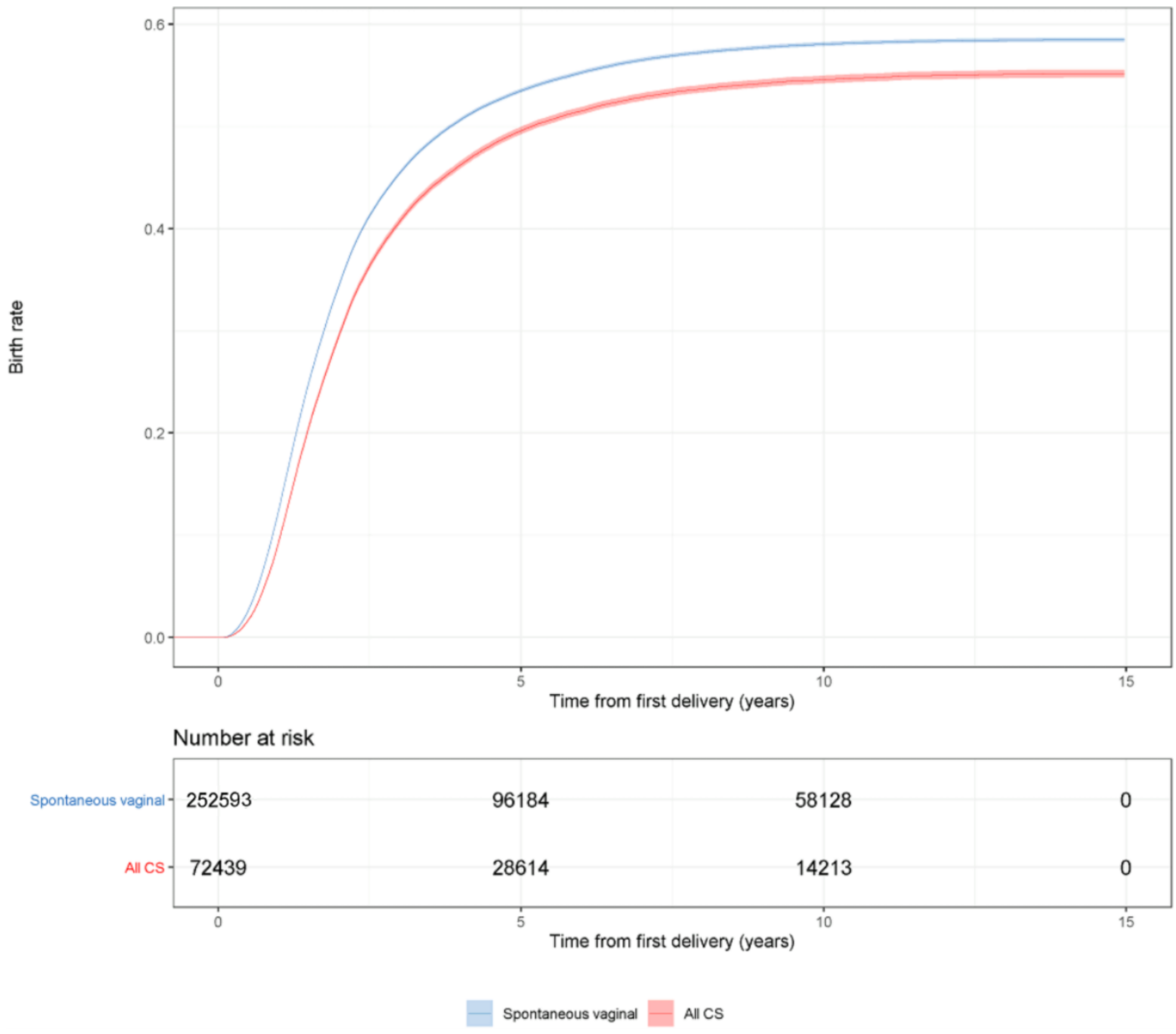

Supplementary figure 1: Kaplan-Meier survival curve with 95% confidence intervals for the event of women becoming pregnant again after their first delivery. Women with CS overall in 1st pregnancy delivery were compared to women who had spontaneous vaginal deliveries (including breech).
