# Supplementary material for: Delivery mode and subsequent birth rate: A nationwide register‐based analysis in Finland
Source: Int J Gynaecol Obstet. 2024 Oct 27;168(3):1161–70. doi: 10.1002/ijgo.15982 (PMC11823355; doi:10.1002/ijgo.15982)
Supplement: Supplementary file 2 — Table S1. [file IJGO-168-1161-s001.pdf]

Supplementary table 1: Categorization of the socioeconomic status and total number of patients in each class found in the Medical Birth Register.

Class	Specific socioeconomic status
**Low**	
	Agricultural sole proprietors or workers
	Industrial workers
	Other production workers
	Distribution and service representatives
	Indefinite workers
	Other self-employed persons or sole proprietors
	Unemployed (no profession)
	Unemployed (profession coded separately)
	Long-term unemployed
	Retired persons
**Middle**	
	Junior employees in work management position
	Junior employees in independent office work
	Junior employees in unindependent office work
	Other indefinite junior employees
**High**	
	Senior employees in leadership position
	Senior employees in design and research assignments
	Senior employees working in teaching positions
	Other indefinite senior employees
**Miscellaneous**	
	Homemaker (full-time taking care for children)
	Students
	Entrepreneurs
	Status coded as unknown
**Missing**	Not registered
